# Antibacterial Activity of Leukocyte- and Platelet-Rich Plasma: An* In Vitro* Study

**DOI:** 10.1155/2018/9471723

**Published:** 2018-06-27

**Authors:** Agata Cieślik-Bielecka, Tadeusz Bold, Grzegorz Ziółkowski, Marcin Pierchała, Aleksandra Królikowska, Paweł Reichert

**Affiliations:** ^1^Department of Maxillofacial Surgery, Provincial Hospital, Plac Medyków 1, 41-200 Sosnowiec, Poland; ^2^Department and Clinic of Orthopaedics, Medical University of Silesia, Plac Medyków 1, 41-200 Sosnowiec, Poland; ^3^Department of Microbiology, Provincial Hospital, Plac Medyków 1, 41-200 Sosnowiec, Poland; ^4^The College of Physiotherapy in Wroclaw, Kościuszki 4, 50-038 Wroclaw, Poland; ^5^Division of Sports Medicine, Department of Physiotherapy, Faculty of Health Sciences, Wroclaw Medical University, Bartla 5, 51-618 Wroclaw, Poland

## Abstract

The aim of the study was to investigate the leukocyte- and platelet-rich plasma (L-PRP) antimicrobial activity. The studied sample comprised 20 healthy males. The L-PRP gel, liquid L-PRP, and thrombin samples were tested* in vitro* for their antibacterial properties against selected bacterial strains using the Kirby-Bauer disc diffusion method. Two types of thrombin were used (autologous and bovine). Zones of inhibition produced by L-PRP ranged between 6 and 18 mm in diameter. L-PRP inhibited the growth of* Staphylococcus aureus* (MRSA and MSSA strains) and was also active against* Enterococcus faecalis* and* Pseudomonas aeruginosa*. There was no activity against* Escherichia coli* and* Klebsiella pneumoniae*. The statistically significant increase of L-PRP antimicrobial effect was noted with the use of major volume of thrombin as an activator. Additionally, in groups where a bovine thrombin mixture was added to L-PRP the zones of inhibition concerning MRSA,* Enterococcus faecalis*, and* Pseudomonas aeruginosa* were larger than in the groups with autologous thrombin. Based on the conducted studies, it can be determined that L-PRP can evoke* in vitro* antimicrobial effects and might be used to treat selected infections in the clinical field. The major volume of thrombin as an activator increases the strength of the L-PRP antimicrobial effect.

## 1. Introduction

Due to their numerous important properties, platelets started to be used in the contemporary medicine as a strategy to stimulate tissue healing, as an autologous cell and plasma fraction extracted from the peripheral blood [1]. Most authors believe that blood concentrate activation and the release of platelet-derived growth factors lead to the acceleration of the wound healing process [1–3]. Moreover, several authors started to argue for the key role of fibrin architecture and leukocyte content of these preparations [4, 5].

Despite advances in surgical techniques, antibiotic administration, and new biomaterials, the treatment of infected wounds continues to be associated with a high rate of complications and belongs to the greatest challenges for surgeons. Previous* in vitro* investigations showed that leukocyte- and platelet-rich plasma (L-PRP) gel is active against selected bacterial strains [6–8]. Some authors also reported clinical observations about a decreasing number of infections and the induction of healing processes after L-PRP use in orthopedics [9], trauma [10], maxillofacial [11], and cardiac surgery [12].

In recent years the discussion was enriched by a few studies focusing on platelet and leukocyte concentrates and their antimicrobial properties related to the presence of natural antibacterial peptides [13]. Antibacterial peptides (HDP: Host Defense Peptides) are produced by macrophages and epithelial cells as well as neutrophils and thrombocytes and are one of the most important elements shaping the natural human immunity. These proteins are synthesized with the use of lipopolysaccharides from the cellular membrane of gram-negative bacteria and display a wide spectrum of activity against gram-positive and gram-negative bacteria as well as viruses and fungi [14–16].

Combining osteoinductive properties with antimicrobial activity and wide use might be a turning point in the treatment of infectious soft tissue and bone defects. However, the mechanism of the antimicrobial effect is not yet fully understood [6–8]. Therefore, the aim of the study was to investigate the leukocyte- and platelet-rich plasma (L-PRP) antimicrobial activity.

## 2. Material and Methods

The experiment gained approval of the local ethics committee and was conducted according to the ethics guidelines and principles of the Declaration of Helsinki. All the participants that took part in the study were informed about the purpose and method of research and signed their informed consent form to participate in the study.

The study group consisted of 20 healthy male volunteers from 30 to 35 years. All subjects were infection-free and at the time of the study none of them had been taking any antibiotics or other medicines during the past three months.

### 2.1. Preparation of L-PRP

For the study, 54 ml of whole blood was collected from each participant into a syringe containing 6.5 ml of citrate dextrose solution (ACD-A). Whole blood was drawn into a sterile tube and centrifuged for 15 minutes at 3200 RPM with 1600 g-force (GPS II Platelet Concentration System, Biomet, Warsaw, USA). This resulted in blood separation into its three basic components: red blood cells, L-PRP sometimes referred to as “buffy coat”, and acellular plasma (AP). Because of differential densities, the red blood cell layer is formed at the lowest level, the L-PRP comprises the middle layer, and AP forms the upper layer. The cell separator system separates each layer from the less dense to the more dense one; therefore it separates AP first and L-PRP second, leaving the residual red blood cells. Subsequently, the AP component was removed into a 30 ml syringe. Next the tube was shaken vigorously for 30 seconds to suspend platelets. Then, 6 ml of L-PRP from the tube was separated and stored at room temperature.

### 2.2. Hematology Analysis

The 2 ml of L-PRP was collected from each sample into a syringe after centrifugation process for hematology analysis. The samples were processed under standardized and optimized conditions within less than 1.5 hours after collection. Acquisition of data was performed in 8-color 3-laser flow cytometer BD FACSCanto II™ (Becton-Dickinson Immunocytometry Systems, San Jose, CA, USA). The data were acquired and analyzed with Diva software (Becton-Dickinson Immunocytometry Systems, San Jose, CA, USA).

### 2.3. Preparation of Thrombin

1.5 ml of autologous thrombin from single donor plasma was prepared using Thrombin Processing Device (TPD™; ThermoGenesis, Rancho Cordova, USA). Plasma from each participant and thrombin reagent consisting of calcium chloride and ethanol was added to the TPD reaction chamber and the contents were mixed and incubated at room temperature. The TPD was then agitated to break any formed fibrinogen clots and the produced thrombin was ready for harvest. Also 1.5 ml of 1600 U/ml bovine thrombin powder (Bio Trombina® 400; Biomed, Lublin, Poland) in a 10% calcium chloride solution was prepared at room temperature. Both autologous thrombin and bovine thrombin were used immediately after their preparation.

### 2.4. In Vitro Antimicrobial Activity Evaluation

The* in vitro* antimicrobial susceptibility testing was performed according to the European Committee of Antimicrobial Susceptibility Testing, EUCAST. The reference strains were derived from the American Type Culture Collection, ATCC®, as strains for testing and validating the method.

*In vitro *laboratory susceptibility to L-PRP gel, liquid L-PRP, and thrombin was determined by the Kirby-Bauer disc diffusion method on Mueller-Hinton agar (Becton-Dickinson Cockeysville, Maryland).

Several of the same colonies of bacteria from fresh, 18-hour bacterial cultures were suspended in sterile physiological NaCl solution (0.85%) to obtain a suspension with a density of 0.5 on the McFarland scale, which approximately corresponds to a number from 1-2 x 10 to 8 c.f.u./ml. The density of the suspension was determined nephelometrically using a Becton-Dickinson colorimeter. Agar plates were inoculated with one of the following bacterial strains: methicillin-resistant* Staphylococcus aureus *(MRSA-ATCC 43300), methicillin-sensitive* Staphylococcus aureus *(MSSA-ATCC 25923),* Escherichia coli *(Extended Spectrum Beta Lactamase, ESBL-ATCC 35218),* Escherichia coli *(ATCC 25922),* Klebsiella pneumoniae *(ESBL-ATCC 700603),* Enterococcus faecalis *(ATCC 29212), and* Pseudomonas aeruginosa *(ATCC 27853). After drying (up to 15 minutes), sterile seven paper discs with a diameter of 6 mm with were placed in each plate to the seeded substrate. Each disc was pressed gently, ensuring an even contact with the ground. Before being placed on the plates, the filter paper discs had been coated using separate micropipettes with seven different test compounds, thus forming seven different groups (G) [17].

The following test compounds were used in the particular groups: G1, 20 *μ*l of L-PRP and 5 *μ*l of autologous thrombin (gelatinous mass); G2, 20 *μ*l of L-PRP and 2 *μ*l of autologous thrombin (gelatinous mass); G3, 25 *μ*l of liquid L-PRP; G4, 25 *μ*l of autologous thrombin; G5, 20 *μ*l of L-PRP and 5 *μ*l of bovine thrombin in a calcium chloride solution; G6, 20 *μ*l of L-PRP and 2 *μ*l of bovine thrombin in a calcium chloride solution; G7, 25 *μ*l of bovine thrombin in a calcium chloride solution.

**Figure 1:**
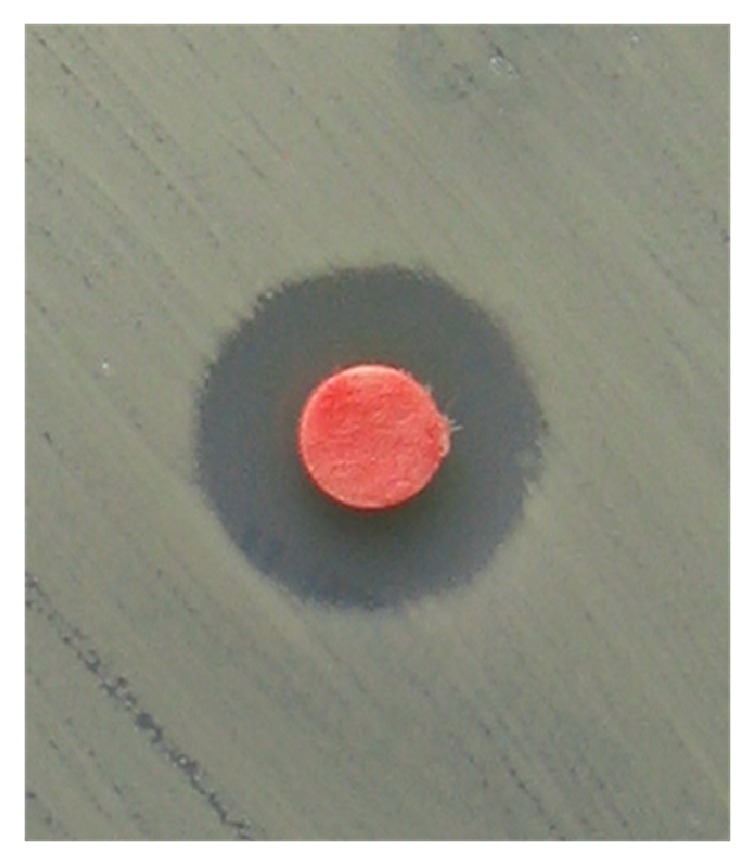
The zone of inhibition measured to determine antimicrobial susceptibility of 20*μ*l L-PRP and 5*μ*l of autologous thrombin against methicillin-resistant* Staphylococcus aureus* (MRSA-ATCC 43300) in participant number 8.

Within 15 minutes, the plates were inserted into the incubator where they were kept at 35°C under aerobic conditions. Baseline antimicrobial activity was assessed after 16 to 18 hours by measuring zones of inhibition across the center of the embedded discs. The diameter of the zone of inhibition was measured including the diameter of the disc and expressed in millimeters. An example of the zone of inhibition was presented in Figure 1.

### 2.5. Statistical Analysis

Statistical analysis was performed using Statistica for Windows software, version 8.0 (Statsoft, Krakow, Poland). The arithmetic means (x) and standard deviations (SD, ±) in the particular studied groups were calculated for the size of inhibition zones. The t-test for two independent samples was performed to compare the inhibition zones between the particular groups. Probability values* p* ≤ 0.05 were considered significant.

## 3. Results

Hematology analysis revealed that average total white blood cells count in the L-PRP amounted to 29.79±5.89 k/*μ*l. The average platelets count in the L-PRP amounted to 952.95±174.25 k/*μ*l.

Table 1 shows the results of baseline tests for L-PRP gel, liquid L-PRP, and thrombin-coated discs against various bacteria. Zones of inhibition produced by L-PRP ranged from 6 to 18 mm in diameter. No antibacterial activity was observed in the case of thrombin groups (G5 and G7).

### 3.1. Methicillin-Resistant* Staphylococcus aureus *(MRSA)

L-PRP gel inhibited the growth of MRSA in all cases (Figure 1). An average zone for L-PRP mixed with 5 *μ*l of autologous (G1) and 5*μ*l of bovine thrombin (G5) was 12.8 and 15.2 mm, respectively (Table 2). The differences in antimicrobial activity between the groups were highly significant (*p* <0.001) as presented in the Table 3. In G2 and G6 the average inhibition zone was smaller in comparison with G1 and G4, and differences were also significant (*p* <0.001). In 12 plates coated with MRSA a zone of inhibition around the disc containing liquid L-PRP (*p<0.001*) was observed.

### 3.2. Methicillin-Sensitive* Staphylococcus aureus *(MSSA)

L-PRP gel inhibited the growth of MSSA in all cases in G1. However, in G2 only in 8 cases and in G6 in 4 cases antimicrobial microbial effects were noted. No activity in the liquid L-PRP group (G3) was observed. In the G5 L-PRP gel inhibited the growth of MSSA in all cases and the differences in antibacterial activity between G1 and G5 were not statistically significant (*p* = 0.199).

### 3.3. Enterococcus faecalis

The average zone for L-PRP mixed with autologous thrombin (G1 and G2) was 11.15 and 7.70 mm, respectively, and the differences between the groups were significant (Table 3). The antimicrobial effect was observed in 19 out of 20 samples in the G3 (Table 1). A zone of inhibition around the discs coated with* Enterococcus faecalis* with L-PRP and bovine thrombin in all cases in G5 and in 15 cases in G6 was noted, and it was significantly stronger than in the groups with autologous thrombin usage, G1 (*p* <0.001) and G2 (*p* <0.001), as presented in the Table 3.

### 3.4. Pseudomonas aeruginosa

Antimicrobial activity against* Pseudomonas aeruginosa* was similar to that against* Enterococcus faecalis*. However, microbicidal effects were weaker than in the previous bacteria; e.g., in the G6 activity was observed in only 4 plates coated with* Pseudomonas aeruginosa* (*p* = 0.042).

### 3.5. Escherichia coli (ESBL), Escherichia coli, and Klebsiella Pneumoniae (ESBL)

No activity against* Escherichia coli* (2 strains) and* Klebsiella pneumoniae* was detected.

## 4. Discussion

The present study confirmed the* in vitro* microbicidal effect of L-PRP against methicillin-resistant* Staphylococcus aureus*, methicillin-sensitive* Staphylococcus aureus, Enterococcus faecalis,* and* Pseudomonas aeruginosa. *No activity of L-PRP was noted against* Klebsiella pneumoniae* and both studied strains of* Escherichia coli*. The major volume of thrombin as an activator increased the strength of the L-PRP antimicrobial effect. Additionally, in case of methicillin-resistant* Staphylococcus aureus* and methicillin-sensitive* Staphylococcus aureus*, greater antimicrobial effect of L-PRP with the bovine thrombin as an activator in comparison to the autologous thrombin was found. The autologous thrombin usage as an activator was more efficient in comparison to the bovine thrombin in case of* Enterococcus faecalis* and* Pseudomonas aeruginosa*.

Platelet concentrates for topical and infiltrative use are defined as blood extracts obtained after various processing of a whole blood sample, mostly through centrifugation [18]. Based on the presence of a cell content and the fibrin architecture, the products are divided into four main families including Pure Platelet-Rich Plasma (P-PRP), also known as Leukocyte-Poor Platelet-Rich Plasma; leukocyte- and platelet-rich plasma (L-PRP); Pure Platelet-Rich Fibrin (P-PRF); and Leukocyte- and Platelet-Rich Fibrin (L-PRF) [1]. Platelet concentrates preparations have been used since 1970s, and they become popular in 1990s. Since then, different protocols were emerging to prepare PRP including commercial systems [17]. Due to the variety of protocols for preparing PRP [17], it is difficult to assess its therapeutic effectiveness. Thrombin is naturally delivered serine protease and it is a critical component for clot formation. Topical thrombin types have been applied to variety of anatomic sites and have been used in many different procedures. The lack of consensus regarding a standard PRP preparation inhibits any comparisons of treatment efficacy obtained by different research groups. Another issue is the inclusion or not of leukocytes as PRP with leukocytes (L-PRP) presents different biologic activity, which could modify the therapeutic effect [19]. However, autologous platelet concentrates for topical and infiltrative are successfully adopted in a variety of medical fields, and they are also believed to exhibit antimicrobial properties. To date, the antimicrobial effect of platelet concentrates against* Staphylococcus aureus* [6, 7, 20, 21],* Escherichia coli* [6, 20],* Pseudomonas aeruginosa* [20],* Klebsiella pneumoniae* [20], and* Enterococcus* faecalis has been reported [22]. Tohidnezhad et al. (2011 and 2012) noted antimicrobial action of platelet concentrates, for example, PRP against E*scherichia coli, Bacillus megaterium, Pseudomonas aeruginosa, Enterococcus faecalis, Klebsiella pneumoniae*, and* Proteus mirabilis* [23, 24]. Drago et al. (2013) reported that P-PRP inhibited the growth of* Enterococcus faecalis, Candida albicans, Streptococcus agalactiae,* and* Streptococcus oralis* but not of* Pseudomonas aeruginosa* [22]. Other authors found no activity of the platelet-rich gel against* Klebsiella pneumoniae* [6],* Enterococcus faecalis* [6], and* Pseudomonas aeruginosa* [6]. In the present study the in vitro microbicidal effect of L-PRP against methicillin-resistant* Staphylococcus aureus*, methicillin-sensitive* Staphylococcus aureus*,* Enterococcus faecalis*, and* Pseudomonas aeruginosa *was noted. There was no activity noted against* Klebsiella pneumoniae* and both studied strains of* Escherichia coli*. Nevertheless, the discrepancies in the evaluation of the antibacterial effect of platelet concentrates may result from the terminology concerning platelet concentrates usage in the studies evaluating their antimicrobial properties which is unclear; therefore the issue remains debatable.

There is also lack of consensus regarding the choice of the best activator and whether PRP should be used with or without activation in clinical practice [25]. The most commonly used ones are both human- and bovine-derived thrombin products. Despite their popular use and rare reports of any adverse reactions, bovine thrombin products are linked to the development of antibodies against human coagulation proteins [26, 27]. According to that, human-derived thrombin formulations and recombinant protein thrombin formulations have been developed. Because of low incidence of adverse event and cost-effectiveness the bovine thrombin products are still widely used. This present study showed that major volume of thrombin as an activator increases the strength of the L-PRP antimicrobial effect, with statistical significance. Interestingly, the present study showed that some of the bacterial strains tested were more sensitive to L-PRP in combination with bovine thrombin and others to L-PRP in combination with human-delivered thrombin. This is an interesting and not yet analyzed aspect of research on the antibacterial effect of L-PRP and this topic should be extended in the future.

The mechanism of L-PRP antimicrobial activity is unknown and we did not find any reports on antibacterial properties of L-PRP in the available literature which were correlated with platelet and leukocyte amount.

However, the existing evidence suggests that platelets have multiple functional attributes in antimicrobial host defense [28–30]. These functions are generated by antimicrobial oxygen metabolites including superoxide, hydrogen peroxide, and hydroxyl free radicals. Moreover, platelets interact directly with microorganisms, contribute to the clearance of pathogens from the bloodstream, and participate to a significant extent in antibody-dependent cell cytotoxicity against microbial pathogens. Since the early studies, numerous investigators have sought to isolate platelet-specific antimicrobial molecules from animal and human platelets [29–32].

At least seven human platelet antimicrobial proteins (HPAPs) are known: fibrinopeptide A and fibrinopeptide B, thymosin beta 4, platelet basic protein, connective tissue-activating protein 3, RANTES (Regulated on Activation Normal T Cell Expressed and Secreted), and platelet factor 4, which have antibacterial properties acting against methicillin-susceptible* Staphylococcus aureus* (MSSA) and methicillin-resistant* Staphylococcus aureus* (MRSA),* Escherichia coli*,* Candida albicans*, and* Cryptococcus neoformans* [29]. In the available literature there is only a little information on the structure and function of platelet antibacterial proteins.

On the basis of the conducted studies, RANTES protein, together with the platelet factor 4 (PF4), was classified in the group of chemokines, which play a key role in bacterial infections caused by intracellular pathogens. It is believed that the level of RANTES increases in the acute phase of infection in tuberculosis and in* Pneumocystis carinii* infection as well as in gastritis and concomitant Helicobacter pylori infection [33–35]. The presence of the RANTES chemokine was observed in the cerebrospinal fluid in children with viral and bacterial inflammations [33]. Platelet factor 4 is a protein produced in megakaryocytes and stored like many other HPAPs in platelet alpha granules. Its release during the activation of thrombocytes intensifies their aggregation under the influence of ADP* in vitro*. The most important task of the platelet factor 4 is to neutralize heparin-like compounds in the blood plasma and on the surface of endothelium and to decrease the activity of antithrombin III (AT III). It also shows a strong antibacterial activity [36].

Like in the other studies [6], there were cases where no antibacterial activity was observed. Nevertheless, animal studies and human clinical trials have shown the benefit of L-PRP application during surgeries by enhancing soft tissue and bone healing despite concurrent infection. Increasingly, apart from the direct antimicrobial effect, growth factors, which are released in high concentrations during platelet and leukocyte aggregation, might also exert clinical antibacterial effects by the induction of neovascularization at the wounded site, which supports self-clearing [37].

An interesting development of research on the antibacterial properties of L-PRP would be to assess the local use of L-PRP in the treatment of, for example, oral infection by* Staphylococcus aureus* in patients affected by white sponge nevus [38]. Also in the future it would be highly interesting to investigate the influence of age-related stress on the overall ability of L-PRF to be biologically active against bacteria, as there are many correlations among stress and biological variation at different levels of activity [39].

## 5. Conclusions

Based on the conducted studies, it can be determined that L-PRP can evoke* in vitro* antimicrobial effects and might be used to treat selected infections in the clinical field. The major volume of thrombin as an activator increases the strength of the L-PRP antimicrobial effect. The use of platelet concentrate technologies for the collection and use of natural antimicrobial agents of the body is a very interesting approach.

## Figures and Tables

**Table 1:** Zones of inhibition determined by L-PRP and thrombin.

	G1 20*µ*l L-PRP + 5*µ*l autologous thrombin							G2 20*µ*l L-PRP + 2*µ*l autologous thrombin							G3 25 *µ*l L-PRP							G4 25*µ* autologous thrombin							G5 20*µ*l L-PRP + 5*µ*l bovine thrombin							G6 20*µ*l L-PRP + 2*µ*l bovine thrombin							G7 25*µ*l bovine thrombin						
No	ATCC® 43300	ATCC® 25923	ATCC® 35218	ATCC® 25922	ATCC® 700603	ATCC® 29212	ATCC® 27853	ATCC® 43300	ATCC® 25923	ATCC® 35218	ATCC® 25922	ATCC® 700603	ATCC® 29212	ATCC® 27853	ATCC® 43300	ATCC® 25923	ATCC® 35218	ATCC® 25922	ATCC® 700603	ATCC® 29212	ATCC® 27853	ATCC® 43300	ATCC® 25923	ATCC® 35218	ATCC® 25922	ATCC® 700603	ATCC® 29212	ATCC® 27853	ATCC® 43300	ATCC® 25923	ATCC® 35218	ATCC® 25922	ATCC® 700603	ATCC® 29212	ATCC® 27853	ATCC® 43300	ATCC® 25923	ATCC® 35218	ATCC® 25922	ATCC® 700603	ATCC® 29212	ATCC® 27853	ATCC® 43300	ATCC® 25923	ATCC® 35218	ATCC® 25922	ATCC® 700603	ATCC® 29212	ATCC® 27853

1	12	10	6	6	6	10	10	8	6	6	6	6	7	7	6	6	6	6	6	7	6	6	6	6	6	6	6	6	15	9	6	6	6	8	7	10	6	6	6	6	7	6	6	6	6	6	6	6	6

2	12	10	6	6	6	11	11	8	6	6	6	6	8	7	7	6	6	6	6	8	7	6	6	6	6	6	6	6	15	10	6	6	6	9	8	9	6	6	6	6	7	6	6	6	6	6	6	6	6

3	12	11	6	6	6	11	11	8	7	6	6	6	7	8	7	6	6	6	6	7	7	6	6	6	6	6	6	6	15	10	6	6	6	9	8	9	6	6	6	6	6	6	6	6	6	6	6	6	6

4	13	11	6	6	6	12	11	9	7	6	6	6	7	7	7	6	6	6	6	7	7	6	6	6	6	6	6	6	16	10	6	6	6	10	7	9	7	6	6	6	7	6	6	6	6	6	6	6	6

5	12	10	6	6	6	11	10	8	7	6	6	6	8	6	6	6	6	6	6	7	6	6	6	6	6	6	6	6	15	8	6	6	6	9	6	10	6	6	6	6	7	6	6	6	6	6	6	6	6

6	13	10	6	6	6	10	10	8	6	6	6	6	8	7	6	6	6	6	6	8	7	6	6	6	6	6	6	6	15	9	6	6	6	9	7	10	6	6	6	6	8	6	6	6	6	6	6	6	6

7	12	11	6	6	6	11	11	8	6	6	6	6	8	7	6	6	6	6	6	8	6	6	6	6	6	6	6	6	13	9	6	6	6	8	7	10	6	6	6	6	7	6	6	6	6	6	6	6	6

8	13	11	6	6	6	10	11	8	6	6	6	6	8	7	6	6	6	6	6	7	6	6	6	6	6	6	6	6	15	11	6	6	6	8	8	9	6	6	6	6	7	6	6	6	6	6	6	6	6

9	14	11	6	6	6	11	11	9	7	6	6	6	8	7	7	6	6	6	6	7	7	6	6	6	6	6	6	6	16	18	6	6	6	8	8	10	7	6	6	6	8	7	6	6	6	6	6	6	6

10	12	10	6	6	6	12	11	7	6	6	6	6	8	6	7	6	6	6	6	8	7	6	6	6	6	6	6	6	15	9	6	6	6	9	8	9	6	6	6	6	7	6	6	6	6	6	6	6	6

11	12	10	6	6	6	11	11	7	6	6	6	6	8	7	7	6	6	6	6	8	7	6	6	6	6	6	6	6	14	9	6	6	6	8	8	9	6	6	6	6	8	6	6	6	6	6	6	6	6

12	13	12	6	6	6	12	12	8	7	6	6	6	8	8	7	6	6	6	6	8	7	6	6	6	6	6	6	6	16	11	6	6	6	9	8	8	6	6	6	6	8	6	6	6	6	6	6	6	6

13	11	10	6	6	6	10	10	7	6	6	6	6	6	6	6	6	6	6	6	6	6	6	6	6	6	6	6	6	14	10	6	6	6	9	8	8	6	6	6	6	6	6	6	6	6	6	6	6	6

14	12	10	6	6	6	11	11	8	6	6	6	6	7	7	7	6	6	6	6	7	7	6	6	6	6	6	6	6	16	9	6	6	6	8	9	9	6	6	6	6	7	6	6	6	6	6	6	6	6

15	13	11	6	6	6	12	11	8	6	6	6	6	8	6	6	6	6	6	6	7	6	6	6	6	6	6	6	6	17	10	6	6	6	10	8	8	6	6	6	6	7	7	6	6	6	6	6	6	6

16	15	13	6	6	6	13	13	9	7	6	6	6	8	8	7	6	6	6	6	8	7	6	6	6	6	6	6	6	17	12	6	6	6	11	10	9	7	6	6	6	6	7	6	6	6	6	6	6	6

17	16	13	6	6	6	13	13	10	7	6	6	6	8	8	7	6	6	6	6	8	7	6	6	6	6	6	6	6	18	12	6	6	6	10	9	11	7	6	6	6	8	7	6	6	6	6	6	6	6

18	14	12	6	6	6	11	12	9	7	6	6	6	8	7	7	6	6	6	6	7	7	6	6	6	6	6	6	6	15	11	6	6	6	8	9	11	6	6	6	6	7	6	6	6	6	6	6	6	6

19	12	10	6	6	6	10	11	8	6	6	6	6	8	7	6	6	6	6	6	8	7	6	6	6	6	6	6	6	12	9	6	6	6	8	8	9	6	6	6	6	6	6	6	6	6	6	6	6	6

20	13	12	6	6	6	11	12	8	6	6	6	6	8	7	7	6	6	6	6	8	7	6	6	6	6	6	6	6	15	11	6	6	6	10	9	11	6	6	6	6	6	6	6	6	6	6	6	6	6

Values are expressed in millimeters. No, participant serial number. Bacterial strains: *Staphylococcus aureus* ATCC 43300 (MRSA), *Staphylococcus aureus* ATCC 25923 (MSSA), *Escherichia coli* ATCC 35218, *Escherichia coli* ATCC 25922, *Klebsiella pneumoniae* ATCC 700603, *Enterococcus faecalis* ATCC 29212, and *Pseudomonas aeruginosa* ATCC 27853.

**Table 2:** Mean values (x) and standard deviation (SD) of inhibition zone determined by L-PRP and thrombin.

		G1 20*µ*l L-PRP + 5*µ*l autologous thrombin	G2 20*µ*l L-PRP + 2*µ*l autologous thrombin	G3 25 *µ*l L-PRP	G4 25*µ* autologous thrombin	G5 20*µ*l L-PRP + 5*µ*l bovine thrombin	G6 20*µ*l L-PRP + 2*µ*l bovine thrombin	G7 25*µ*l bovine thrombin
ATCC® 43300	x	12.80	8.15	6.60	6.00	18.00	11.00	6.00
	SD	1.20	0.75	0.50	0.00	1.36	0.94	0.00

ATCC® 25923	x	10.90	6.40	6.00±	6.00	18.00	7.00	6.00
	SD	1.02	0.50	0.00	0.00	2.11	0.41	0.00

ATCC® 35218	x	6.00	6.00	6.00	6.00	6.00	6.00	6.00
	SD	0.00	0.00	0.00	0.00	0.00	0.00	0.00

ATCC® 25922	x	6.00	6.00	6.00	6.00	6.00	6.00	6.00
	SD	0.00	0.00	0.00	0.00	0.00	0.00	0.00

ATCC® 700603	x	6.00	6.00	6.00	6.00	6.00	6.00	6.00
	SD	0.00	0.00	0.00	0.00	0.00	0.00	0.00

ATCC® 29212	x	11.15	7.70	7.45	6.00	11.00	8.00	6.00
	SD	0.93	0.57	0.61	0.00	0.91	0.73	0.00

ATCC® 27853	x	11.15	7.00	6.70	6.00	10.00	7.00	6.00
	SD	0.88	0.65	0.47	0.00	0.92	0.41	0.00

The values expressed in millimeters. Bacterial strains: *Staphylococcus aureus* ATCC 43300 (MRSA), *Staphylococcus aureus* ATCC 25923 (MSSA), *Escherichia coli* ATCC 35218, *Escherichia coli* ATCC 25922, *Klebsiella pneumoniae* ATCC 700603, *Enterococcus faecalis* ATCC 29212, and *Pseudomonas aeruginosa* ATCC 27853.

**Table 3:** Comparison of antimicrobial activity between the studied groups.

Studied Group	G1	G2	G3	G4	G5	G6	G7
	methicillin-resistant *Staphylococcus aureus *(MRSA-ATCC® 43300)						

G1	-	≤0.001	<0.001	<0.001	<0.001	<0.001	<0.001

G2	<0.001	-	<0.001	<0.001	<0.001	<0.001	<0.001

G3	<0.001	<0.001	-	<0.001	<0.001	<0.001	<0.001

G4	<0.001	<0.001	<0.001	-	<0.001	<0.001	<0.001

G5	<0.001	<0.001	<0.001	<0.001	-	<0.001	<0.001

G6	<0.001	<0.001	<0.001	<0.001	<0.001	-	<0.001

G7	<0.001	<0.001	<0.001	<0.001	<0.001	<0.001	-

	methicillin-sensitive Staphylococcus aureus (MSSA-ATCC® 25923)						

G1	-	<0.001	<0.001	<0.001	0.199	<0.001	<0.001

G2	<0.001	-	0.002	0.002	<0.001	0.042	0.002

G3	<0.001	0.002	-	AMR	<0.001	0.042	AMR

G4	<0.001	0.002	AMR	-	<0.001	0.042	AMR

G5	0.199	<0.001	<0.001	<0.001	-	<0.001	<0.001

G6	<0.001	0.042	0.042	0.042	<0.001	-	0.042

G7	<0.001	0.002	AMR	AMR	<0.001	0.042	-

	*Enterococcus faecalis *(ATCC® 29212)						

G1	-	<0.001	<0.001	<0.001	<0.001	<0.001	<0.001

G2	<0.001	-	0.021	<0.001	<0.001	<0.001	<0.001

G3	<0.001	0.021	-	<0.001	<0.001	0.025	<0.001

G4	<0.001	<0.001	<0.001	-	<0.001	<0.001	AMR

G5	<0.001	<0.001	<0.001	<0.001	-	<0.001	<0.001

G6	<0.001	<0.001	0.025	<0.001	<0.001	-	<0.001

G7	<0.001	<0.001	<0.001	AMR	<0.001	<0.001	-

	*Pseudomonas aeruginosa *(ATCC® 27853)						

G1	-	<0.001	<0.001	<0.001	<0.001	<0.001	<0.001

G2	<0.001	-	0.030	<0.001	<0.001	<0.001	<0.001

G3	<0.001	0.030	-	<0.001	<0.001	<0.001	<0.001

G4	<0.001	<0.001	<0.001	-	<0.001	0.042	AMR

G5	<0.001	<0.001	<0.001	<0.001	-	<0.001	<0.001

G6	<0.001	<0.001	<0.001	0.042	<0.001	-	0.42

G7	<0.001	<0.001	<0.001	AMR	<0.001	0.042	-

The results are expressed as *p*, level of significance. In some cases *p* was not calculated because of the antimicrobial resistance (AMR) of both groups being compared. G1, L-PRP and 5 *µ*l of autologous thrombin; G2, L-PRP and 2 *µ*l of autologous thrombin; G3, liquid L-PRP; G4, autologous thrombin; G5, L-PRP and 5 *µ*l of bovine thrombin in calcium chloride solution; G6, L-PRP and 2 *µ*l of bovine thrombin in calcium chloride solution; G7; bovine thrombin.

## Data Availability

The data used to support the findings of this study are available from the corresponding author upon request.

## References

[1] Dohan Ehrenfest D. M., Rasmusson L., Albrektsson T. (2009). Classification of platelet concentrates: from pure platelet-rich plasma (P-PRP) to leucocyte- and platelet-rich fibrin (L-PRF). *Trends in Biotechnology*.

[2] Alsousou J., Thompson M., Hulley P., Noble A., Willett K. (2009). The biology of platelet-rich plasma and its application in trauma and orthopaedic surgery: A review of the literature. *The Journal of Bone & Joint Surgery (British Volume)*.

[3] Zumstein M. A., Bielecki T., Dohan Ehrenfest D. M. (2011). The Future of Platelet Concentrates in Sports Medicine: Platelet-Rich Plasma, Platelet-Rich Fibrin, and the Impact of Scaffolds and Cells on the Long-term Delivery of Growth Factors. *Operative Techniques in Sports Medicine*.

[4] Dohan Ehrenfest D. M., Bielecki T., Jimbo R. (2012). Do the fibrin architecture and leukocyte content influence the growth factor release of platelet concentrates? An evidence-based answer comparing a pure Platelet-Rich Plasma (P-PRP) gel and a leukocyte- and Platelet-Rich Fibrin (L-PRF). *Current Pharmaceutical Biotechnology*.

[5] Everts P. A. M., Hoffmann J., Weibrich G. (2006). Differences in platelet growth factor release and leucocyte kinetics during autologous platelet gel formation. *Transfusion Medicine*.

[6] Bielecki T. M., Gazdzik T. S., Arendt J., Szczepanski T., Król W., Wielkoszynski T. (2007). Antibacterial effect of autologous platelet gel enriched with growth factors and other active substances: an in vitro study. *The Journal of Bone & Joint Surgery (British Volume)*.

[7] Moojen D. J., Everts P. A., Schure R. M. (2008). Antimicrobial activity of platelet-leukocyte gel against *Staphylococcus aureus*. *Journal of Orthopaedic Research*.

[8] Mariani E., Filardo G., Canella V. (2014). Platelet-rich plasma affects bacterial growth in vitro. *Cytotherapy*.

[9] Callaghan J. J., Liu S. S., Phruetthiphat O.-A. (2014). The revision acetabulum - Allograft and bone substitutes: Vestigial organs for bone deficiency. *The Bone & Joint Journal*.

[10] Wang H.-F., Gao Y.-S., Yuan T., Yu X.-W., Zhang C.-Q. (2013). Chronic calcaneal osteomyelitis associated with soft-tissue defect could be successfully treated with platelet-rich plasma: a case report. *International Wound Journal*.

[11] Cieślik-Bielecka A., Glik J., Skowroński R., Bielecki T. (2016). Benefit of Leukocyte- and Platelet-Rich Plasma in Operative Wound Closure in Oral and Maxillofacial Surgery. *BioMed Research International*.

[12] Khalafi R. S., Bradford D. W., Wilson M. G. (2008). Topical application of autologous blood products during surgical closure following a coronary artery bypass graft. *European Journal of Cardio-Thoracic Surgery*.

[13] Cieslik-Bielecka A. (2012). Microbicidal properties of Leukocyte- and Platelet-Rich Plasma/Fibrin (L-PRP/L-PRF): new perspectives. *Journal of Biological Regulators & Homeostatic Agents*.

[14] Linde A., Ross C. R., Davis E. G., Dib L., Blecha F., Melgarejo T. (2008). Innate immunity and host defense peptides in veterinary medicine. *Journal of Veterinary Internal Medicine*.

[15] Scott M. G., Hancock R. E. (2000). Cationic Antimicrobial Peptides and Their Multifunctional Role in the Immune System. *Critical Reviews™ in Immunology*.

[16] Šíma P., Trebichavský I., Sigler K. (2003). Nonmammalian Vertebrate Antibiotic Peptides. *Folia Microbiologica*.

[17] Amable P. R., Carias R. B. V., Teixeira M. V. T. (2013). Platelet-rich plasma preparation for regenerative medicine: optimization and quantification of cytokines and growth factors. *Stem Cell Research & Therapy*.

[18] Bielecki T., Dohan Ehrenfest D. M. (2012). Platelet-Rich Plasma (PRP) and Platelet-Rich Fibrin (PRF): surgical adjuvants, preparations for in situ regenerative medicine and tools for tissue engineering. *Current Pharmaceutical Biotechnology*.

[19] Perez A. G., Lana J. F. S., Rodrigues A. A., Luzo A. C. M., Belangero W. D., Santana M. H. A. (2014). Relevant aspects of centrifugation step in the preparation of platelet-rich plasma. *ISRN Hematology*.

[20] Burnouf T., Chou M.-L., Wu Y.-W., Su C.-Y., Lee L.-W. (2013). Antimicrobial activity of platelet (PLT)-poor plasma, PLT-rich plasma, PLT gel, and solvent/detergent-treated PLT lysate biomaterials against wound bacteria. *Transfusion*.

[21] Chen L., Wang C., Liu H., Liu G., Ran X. (2013). Antibacterial effect of autologous platelet-rich gel derived from subjects with diabetic dermal ulcers in vitro. *Journal of Diabetes Research*.

[22] Drago L., Bortolin M., Vassena C., Taschieri S., del Fabbro M. (2013). Antimicrobial activity of pure platelet-rich plasma against microorganisms isolated from oral cavity. *BMC Microbiology*.

[23] Tohidnezhad M., Varoga D., Wruck C. J. (2012). Platelets display potent antimicrobial activity and release human beta-defensin 2. *Platelets*.

[24] Tohidnezhad M., Varoga D., Podschun R. (2011). Thrombocytes are effectors of the innate immune system releasing human beta defensin-3. *Injury*.

[25] Huber S. C. (2016). In vitro study of the role of thrombin in platelet rich plasma (PRP) preparation: utility for gel formation and impact in growth factors release. *Journal of Stem cells and Regenerative Medicine*.

[26] Ortel T. L., Mercer M. C., Thames E. H., Moore K. D., Lawson J. H. (2001). Immunologic impact and clinical outcomes after surgical exposure to bovine thrombin. *Annals of Surgery*.

[27] Streiff M. B., Ness P. M. (2002). Acquired FV inhibitors: a needless iatrogenic complication of bovine thrombin exposure.. *Transfusion*.

[28] Weksler B. B., Nachman R. L. (1971). Rabbit platelet bactericidal protein. *The Journal of Experimental Medicine*.

[29] Yeaman M. R. (1997). Purification and in vitro activities of rabbit platelet microbicidal proteins. *Infect Immun*.

[30] Krijgsveld J., Zaat S. A. J., Meeldijk J. (2000). Thrombocidins, microbicidal proteins from human blood platelets, are C-terminal deletion products of CXC chemokines. *The Journal of Biological Chemistry*.

[31] Dziarski R., Gupta D. (2006). Mammalian PGRPs: Novel antibacterial proteins. *Cellular Microbiology*.

[32] Tang Y.-Q., Yeaman M. R., Selsted M. E. (2002). Antimicrobial peptides from human platelets. *Infection and Immunity*.

[33] Henriquet C., Gougat C., Combes A., Lazennec G., Mathieu M. (2007). Differential regulation of RANTES and IL-8 expression in lung adenocarcinoma cells. *Lung Cancer*.

[34] Huang K., Wang C., Lin C., Kuo H. (2014). NF-*κ*B repressing factor downregulates basal expression and mycobacterium tuberculosis induced IP-10 and IL-8 synthesis via interference with NF-*κ*B in monocytes. *Journal of Biomedical Science*.

[35] Lei G.-S., Zhang C., Lee C.-H. (2015). Myeloid-derived suppressor cells impair alveolar macrophages through PD-1 receptor ligation during Pneumocystis pneumonia. *Infection and Immunity*.

[36] Maurer A.-M., Zhou B., Han Z. C. (2006). Roles of platelet factor 4 in hematopoiesis and angiogenesis. *Growth Factors*.

[37] Cieslik-Bielecka A., Gazdzik T. S., Bielecki T. M., Cieslik T. (2007). Why the platelet-rich gel has antimicrobial activity?. *Oral Surgery, Oral Medicine, Oral Pathology, Oral Radiology, and Endodontology*.

[38] Marrelli M., Tatullo M., Dipalma G., Inchingolo F. (2012). Oral infection by staphylococcus aureus in patients affected by white sponge nevus: A description of two cases occurred in the same family. *International Journal of Medical Sciences*.

[39] Marrelli M., Gentile S., Palmieri F., Paduano F., Tatullo M. (2014). Correlation between surgeon's experience, surgery complexity and the alteration of stress related physiological parameters. *PLoS ONE*.

